# Training for Radiographic Assessment of Scoliosis: A Pilot Educational Study

**DOI:** 10.3390/jcm14030911

**Published:** 2025-01-30

**Authors:** Mirko Filippetti, Sofia Tomasello, Rita Di Censo, Angela Modenese, Dalila Scaturro, Giulia Letizia Mauro, Valentina Varalta, Nicola Smania, Alessandro Picelli

**Affiliations:** 1 Neuromotor and Cognitive Rehabilitation Research Center, Section of Physical and Rehabilitation Medicine, Department of Neurosciences, Biomedicine and Movement Sciences, University of Verona, 37134 Verona, Italy; mirko.filippetti@univr.it (M.F.); sofia.tomasello@studenti.univr.it (S.T.); rita.dicenso@univr.it (R.D.C.); valentina.varalta@univr.it (V.V.); nicola.smania@univr.it (N.S.); 2 Canadian Advances in Neuro-Orthopedics for Spasticity Consortium (CANOSC), Kingston, ON K7K 1Z6, Canada; 3 Department of Neurosciences, University Hospital of Verona, 37126 Verona, Italy; angela.modenese@aovr.veneto.it; 4 Department of Surgical, Oncological and Stomatological Disciplines, University of Palermo, 90127 Palermo, Italy; dalila.scaturro@unipa.it (D.S.); giulia.letiziamauro@unipa.it (G.L.M.)

**Keywords:** education, internship and residency, spine

## Abstract

**Background**: Given the prevalence of scoliosis, it is essential for residents in Physical and Rehabilitation Medicine to develop proficiency in evaluating spinal radiographs, particularly in measuring the Cobb angle. This education and training study aimed to define the minimum educational requirements for residents to achieve competency in Cobb angle measurement with acceptable inter- and intra-operator variability, as reported in the literature. **Methods**: In this ethics-approved study, Physical and Rehabilitation Medicine residents measured Cobb angles and the Risser index using specialized software on 30 different spinal X-rays, with oversight to ensure consistency and eliminate bias. **Results**: The data revealed that 44.4% of residents achieved the target accuracy for primary Cobb angles (<3.6°), but only 11.1% did so for secondary curves. For intra-operator error, 88.9% of residents met the target for primary curves, but only 11.1% achieved the target for secondary curves. The Risser index showed minimal deviation across all participants. **Conclusions**: These findings highlight that mastering Cobb angle measurement is challenging and generally requires more than 20 different measurements for inter-operator reliability and over 30 repetitions for intra-operator reliability, particularly when secondary curves are included.

## 1. Introduction

Among the various postural abnormalities affecting the pelvis, trunk, and shoulders in both the frontal and sagittal planes, scoliosis emerges as a particularly prevalent condition. It serves as an umbrella term for a diverse group of disorders characterized by a three-dimensional deformity of the spine, thorax, and trunk, defined by a Cobb angle exceeding 10° [1]. Scoliosis affects 2–3% of the population, with females being more prone to progression despite similar incidence rates between genders (1.3:1) [2]. Approximately 80% of scoliosis cases are idiopathic, while the remaining 20% are attributed to secondary causes [2].

The current clinical guidelines emphasize measuring the Cobb angle on a full-spine radiograph in the frontal plane while the patient is in an upright position as a main component of scoliosis management [3]. Additionally, the Risser index is commonly assessed in scoliosis patients as an indirect radiographic indicator of skeletal maturity and growth status [2].

Introduced in 1966 by the Scoliosis Research Society (SRS), the Cobb angle remains a fundamental metric for diagnosing and monitoring scoliosis progression [4,5]. Despite technological advancements, Cobb angle measurements are still predominantly performed manually on full-length standing spinal radiographs [6,7]. Initially conducted on analog radiographs, these measurements are now increasingly performed digitally using specialized software. On traditional radiographs, the inter-operator error for Cobb angle measurements typically ranges from 6 to 7 degrees, whereas computer-assisted methods have significantly enhanced accuracy, reducing error rates to between 1.22 and 3.60 degrees [8,9,10].

The up-to-date literature indicates that intra-observer and inter-observer reliability for Cobb angle measurements is consistent among medical professionals involved in scoliosis management, including PGY-3 orthopedic residents, orthopedic surgeons, and radiologists [11]. Also, Physical and Rehabilitation Medicine (PRM) physicians play a key role in the diagnosis and conservative management of spine disorders, including scoliosis. According to the core curriculum for PRM residents outlined by the International Society of Physical and Rehabilitation Medicine, the SRS, and the Italian Ministry of Education and Merit, proficiency in interpreting spinal radiographs for scoliosis assessment is considered an essential skill [12,13,14]. However, despite the emphasis placed on these competencies, specific educational benchmarks for achieving mastery in this area remain undefined. Furthermore, there is a lack of data addressing the relationship between the experience level of observers in PRM settings and their measurement accuracy. To address this gap, this pilot study aims to determine the minimum number of computer-assisted radiograph measurements required for PRM residents to achieve error rates consistent with those deemed acceptable in the literature.

## 2. Materials and Methods

This single-center pilot education and training study, approved by the local ethics committee (Comitato di Approvazione della Ricerca sulla Persona; approval number 04.R1/2023), involved first-year PRM residents from the University of Verona in Italy. Participants were required to have no prior experience in X-ray evaluation of patients with vertebral dysmorphism and to provide informed consent.

Included residents were given access to the 2016 SOSORT guidelines for study [2]. Following a two-week preparatory period, a two-hour group session was conducted under the guidance of a clinical tutor (a PRM specialist with over five years of experience in scoliosis evaluation). During this session, the tutor demonstrated practical techniques for measuring the Cobb angle and assessing the Risser index. Each resident performed two measurement attempts on two different radiographs under the tutor’s supervision. Additionally, residents were introduced to Horos (v. 3.3.6), a free, open-source medical image viewer used for performing measurements.

This study utilized a set of radiographs from 30 consecutive patients admitted to our clinical unit between August and December 2023. The clinical tutor measured all 30 radiographs once and performed a second measurement on 10 randomly selected radiographs to assess intra-operator variability. Each resident completed a total of 40 measurements: 20 evaluations on different radiographs and 20 repeated evaluations of 10 randomly selected radiographs. This design meant that 10 radiographs were measured three times. Organizational aspects, including the scheduling of sessions, were overseen by a separate medical doctor serving as the coordinator. Sessions were spaced at least 10 days apart, and residents measured and re-measured the selected radiographs during these sessions. Each measurement was conducted in the presence of the coordinator, who was responsible for loading and anonymizing the X-rays using a dedicated software function, ensuring the process was conducted without external interference. Measurements focused on recording the Cobb angle for both primary and, where applicable, secondary curves, as well as the Risser index.

## 3. Results

Nine Italian PRM residents, one tutor, and one coordinator participated in this pilot study. The residents collectively performed a total of 360 evaluations (40 per resident). The 30 selected X-rays corresponded to consecutive patients with a mean age of 14.08 years (range: 9–16 years). The mean Cobb angle of the primary curve, as measured by the tutor, was 15.57 ± 8.82 degrees, while the median Risser index was 3 (interquartile range: 2–4). The tutor exhibited an intra-operator variability of 2.71 ± 0.95 degrees for the Cobb angle and demonstrated no variability in the Risser index.

**Figure 1:**
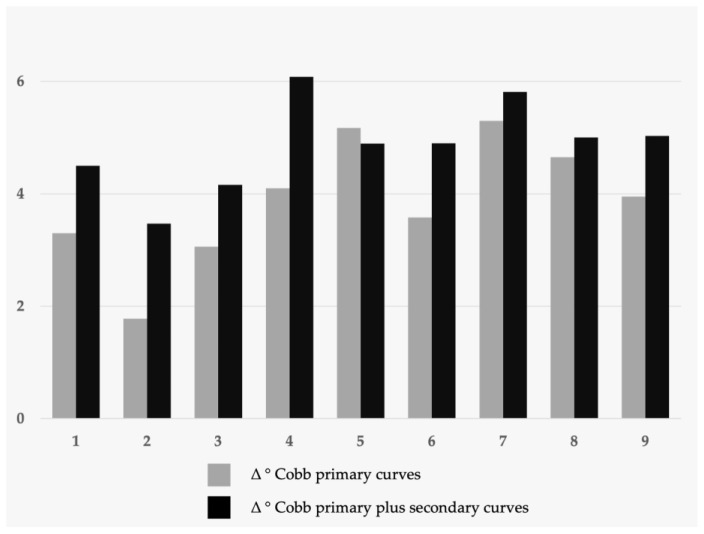
This figure illustrates the average inter-operator error for each resident.

The inter-operator errors (i.e., the discrepancy between each resident’s measurements and those of the tutor) for the primary curves, combined primary and secondary curves, Risser index, and the number of secondary curves misinterpreted in the 20 X-rays assessed by each resident are presented in Table 1 (for added clarity, the mean inter-operator errors are also illustrated in Figure 1).

The variation in intra-operator error for the Cobb angle in primary curves and in both primary and secondary curves is presented in Table 2.

Table 3 details the cumulative inter-operator error (all residents compared to the tutor) for Cobb angle measurements of the primary curves and the combined primary and secondary curves at the fifth, tenth, fifteenth, and twentieth radiographs.

The most common errors identified involved the selection of the right superior and inferior limiting vertebrae and the determination of the right upper or lower endplate. Errors in calculating the Cobb angle on the incorrect side were less frequent.

## 4. Discussion

Despite being emphasized in all the cited core curricula [12,13,14], the importance of achieving proficiency in measuring spinal radiographs in patients with scoliosis lacks a defined minimum number of attempts required for achieving intra- and inter-operator reliability in PRM residents. A detailed analysis of the inter-operator error for the Cobb angle measurements of the primary curves (each resident compared to the tutor; Table 1) revealed that only four out of nine residents (44.4%; Residents 1, 2, 3, and 6) achieved the target (<3.6°). When secondary curves were also considered, only one resident (11.1%; Resident 2) met the target. These findings may seem at odds with the current literature, which reports consistent measurement agreements between PGY-3 orthopedic residents and more experienced professionals such as orthopedic surgeons and radiologists. However, studies have also shown that medical students exhibit poor inter-observer agreement compared to more experienced clinicians [11]. Considering that our sample consisted of first-year PRM residents with no prior experience in X-ray evaluation of scoliosis, our results align more closely with those observed among medical students. This alignment underscores the educational focus of this pilot study: to determine the minimum number of radiograph measurements required for PRM residents to achieve error rates consistent with those deemed acceptable in the literature, thereby contributing to a clinical education framework tailored to their level of expertise.

A notable finding of this study was the high frequency of misinterpretations of secondary curves, likely due to their lower degrees (often <10°). When examining the cumulative deviation between all residents and the tutor (Table 3) and focusing solely on the primary curves, the target variability (<3.6°) was nearly achieved by the 10th and 15th measurements. However, a reversal of this trend was observed by the 20th measurement, undermining what initially appeared to be a learning effect. When secondary curves were included, the target variability was never achieved. Further analysis revealed that four residents failed to identify a secondary curve of 14° on the same radiograph. Excluding this specific error from the dataset produced a trend similar to that observed for the primary curves, emphasizing the critical importance of continued practical training sessions to effectively address and correct procedural errors. The findings related to secondary curves are particularly significant, as S-shaped scoliosis is more complex to manage clinically. It affects multiple spinal segments, presents a worse prognosis, and requires accurate evaluation of both primary and secondary curves for proper management. From a clinical perspective, the accurate assessment of all curves is a key element in effective scoliosis treatment. Additionally, from an educational standpoint, training programs should prioritize the accurate detection and measurement of both primary and secondary curves, as this is essential for equipping residents with the necessary skills for comprehensive scoliosis management.

For intra-operator error in primary curves, eight out of nine residents (88.9%) met the target. However, when secondary curves were included, only one resident (11.1%; Resident 5) remained below the target. This variability may reflect difficulties in accurately identifying the upper and lower limiting vertebrae and tracing lines parallel to the vertebral edges.

The Risser index proved relatively straightforward to learn, with no resident exhibiting significant deviations from the reference data.

This study has several limitations. First, it involved a small sample of PRM residents (nine) and was conducted within a single institution, without accounting for variations in residency programs or access to training resources. Although the pilot nature of the study justifies its limited sample size, this remains a significant constraint on the generalizability of our findings to broader educational contexts. Similarly, reliance on a single tutor for reference measurements is another potential limitation, as it may have introduced bias, even though the tutor demonstrated an acceptable level of intra-operator error. Additionally, the mean Cobb angle of the primary curves was 15.57 degrees, reflecting mild scoliosis. While this might be considered a limitation, we view it as a strength, as more pronounced curves are generally easier to identify and measure. Finally, no intermediate teaching sessions were included to address and correct errors stemming from improper learning, which may have affected the participants’ overall performance.

## 5. Conclusions

Our preliminary results indicate that measuring the Cobb angle is a complex task, often requiring a learning curve exceeding 20 measurements to achieve acceptable inter-operator variability and 30 repetitions for intra-operator consistency, particularly when evaluating secondary curves. Although our observations need to be validated through larger multicenter studies that account for variations in residency programs, we suggest that systematically addressing these challenges requires integrating teaching approaches into residency curricula. These approaches could include periodic intermediate sessions, seminar-based activities, clinical case presentations, and comprehensive training in measurement software. Additionally, innovative tools based on virtual reality or artificial intelligence (AI) may offer valuable support for enhancing this learning process. Recent evidence indicates that AI in musculoskeletal radiology provides lower variability rates and faster evaluations compared to manual measurements [15]. While imaging quality remains a limitation of current AI algorithms, this technology holds significant promise for improving the interpretation of radiographs in scoliosis management, potentially enhancing diagnostic accuracy.

## Figures and Tables

**Table 1:** Inter-operator error (residents vs. tutor).

Resident	Δ Risser	Δ ° Cobb Primary Curves	Δ ° Cobb Primary Plus Secondary Curves	* Incorrect Interpretation of Secondary Curves (n)
1	0.5 (0; 1)	3.30 ± 2.99	4.50 ± 5.72	9
2	0 (0; 1)	1.78 ± 0.94	3.47 ± 3.14	7
3	1 (0; 2.25)	3.06 ± 1.84	4.16 ± 1.84	4
4	0 (0; 1)	4.10 ± 3.93	6.08 ± 5.10	11
5	1 (0; 1)	5.17 ± 4.33	4.89 ± 3.99	2
6	0 (0; 1)	3.58 ± 3.55	4.90 ± 4.89	7
7	0 (0; 1)	5.30 ± 4.84	5.81 ± 5.32	8
8	0 (0; 1)	4.65 ± 5.00	5.00 ± 5.77	7
9	0.5 (0; 1)	3.95 ± 4.49	5.03 ± 5.56	10

Median (Q1; Q3) or mean ± standard deviation where appropriate. ° degrees. * Only secondary curves were misinterpreted.

**Table 2:** Variation in intra-operator error for the Cobb angle.

Resident	Δ ° Cobb Primary Curves	Δ ° Cobb Primary Plus Secondary Curves
1	3.00 (0; 6)	4.72 (0; 23)
2	3.50 (2; 8)	4.56 (2; 20)
3	3.30 (0; 6)	4.44 (0; 7)
4	2.80 (0; 6)	4.59 (0; 21)
5	2.62 (0; 7)	2.50 (0; 8)
6	2.45 (0; 11)	4.11 (0; 15)
7	3.60 (0; 9)	5.57 (0; 14)
8	4.10 (0; 14)	4.82 (0; 14)
9	3.00 (1; 8)	3.68 (1; 9)

The data are presented as mean (minimum; maximum). ° degrees.

**Table 3:** Cumulative inter-operator error (residents vs. tutor).

n	Δ Risser	Δ ° Cobb Primary Curves	Δ ° Cobb Primary Plus Secondary Curves
1–5	0 (0; 1)	3.68 ± 1.52	4.80 ± 1.43
1–10	0 (0; 1)	3.62 ± 1.21	4.75 ± 1.27
1–15	0 (0; 1)	3.42 ± 1.02	4.58 ± 0.90
1–20	0 (0; 1)	3.88 ± 1.11	4.89 ± 0.79

Data are expressed as median (Q1; Q3) or mean ± standard deviation, as appropriate. n: number of X-rays. °: degrees.

## Data Availability

The original contributions presented in this study are included in the article. Further inquiries can be directed to the corresponding author.
